# Awareness, Access and Use of Internet Self-Help Websites for Depression by University Students

**DOI:** 10.2196/mental.5311

**Published:** 2016-10-27

**Authors:** Gordana Culjak, Nick Kowalenko, Christopher Tennant

**Affiliations:** ^2^ Discipline of Psychiatry Sydney Medical School University of Sydney Sydney Australia

**Keywords:** depression, awareness, Internet, self-help, university, students, eHealth, health promotion, prevention

## Abstract

**Background:**

University students have a higher prevalence rate of depression than the average 18 to 24 year old. Internet self-help has been demonstrated to be effective in decreasing self-rated measures of depression in this population, so it is important to explore the awareness, access and use of such self-help resources in this population.

**Objective:**

The objective of this study is to explore university students’ awareness, access and use of Internet self-help websites for depression and related problems.

**Methods:**

A total of 2691 university students were surveyed at 3 time points.

**Results:**

When asked about browsing behavior, 69.6% (1494/2146) of students reported using the Internet for entertainment. Most students were not familiar with self-help websites for emotional health, although this awareness increased as they completed further assessments. Most students considered user-friendliness, content and interactivity as very important in the design of a self-help website. After being exposed to a self-help website, more students reported visiting websites for emotional health than those who had not been exposed.

**Conclusions:**

More students reported visiting self-help websites after becoming aware of such resources. Increased awareness of depression and related treatment resources may increase use of such resources. It is important to increase public awareness with the aim of increasing access to targeted strategies for young people.

## Introduction

The purpose of this study is to explore university students’ awareness, access and use of Internet self-help websites for depression and related problems in a sample of university students in the 18 to 24 year age group. This age group has a high prevalence of depression and university students have been found to have an even higher prevalence rate than the average 18 to 24 year old (20% compared with 10%, respectively) [1]. Furthermore, it has been shown that Internet self-help is effective in decreasing self-rated measures of depression in this population, so it is highly relevant to explore the awareness, access and use of such self-help resources in this population.

### Awareness in the General Population

In exploring awareness of depression in the general population it was found that there are a growing number of public health campaigns that aim to increase awareness of depression and related issues (eg, beyondblue). Beyondblue is a National Depression Initiative in Australia that has a large advertising campaign on television, buses, letter box drops etc [2]; such health promotion strategies are common in similar campaigns worldwide [3]. The United States has a National Depression Screening Day which is part of the National Mental Illness Awareness Week that occurs each October [4]. In 1993 the National Public Education Campaign on Clinical Depression was launched and is sponsored by the Mental Health Association and over 100 professional and advocacy groups [5]. The American Medical Association is also working with the National Institute of Mental Health to increase awareness [6].

It has been reported that these campaigns enhance public education and awareness and improve professional recognition and management of depression [7]. Studies have shown that with regards to treatment, people who had sought help were less likely to find family and social support helpful and more likely to believe in medical interventions such as antidepressants. These postal survey participants rated holidays, massages and recreational activities as helpful [8]. This was similar to previous findings that showed prior treatment, higher education and greater episode length predicted treatment-seeking behavior, whereas non-treatment-seekers felt they could handle it themselves and either did not recognize or see depression as serious [9]. Professional treatment was often perceived as helpful, but rarely used [10]. Most people with symptoms of depression and anxiety access “simple self-help interventions”. It is therefore important to explore awareness, access and use of such self-help interventions.

### Awareness in the University Student Population

A Swiss study explored awareness of mental disorders in university students and found a wide variability in mental health literacy. For depression, however, they found that most students recognized the symptoms [11] and concluded that special attention should be paid towards the effects of gender and stereotypes on mental illness.

### Health Information on the Internet

A National Survey in the United States has found that 80% of Internet users look for health information online, and that 21% look for depression, anxiety, stress or mental health information [12]. From the information they obtain on the Internet, 47% of consumers say that their findings influenced their decisions about treatment and care, prepared them to ask more questions or get a second opinion and influenced their decisions about whether or not to visit a doctor [13].

Web-based treatments and education about depression and anxiety have been cited as having the advantage of facilitating broader access for both sufferers who do not seek external help and for consumers who have access to Web-based guidelines [14].

### People Do Not Seek Help

It was found that only 20.1% of 18 to 54 year olds with emotional disorders received treatment between 2001 and 2003 [15]. The National Finnish Health Care Survey revealed that of people suffering from a major depressive episode over the last 12 months, only 31% of men and 25% of women used any type of health care services, with people more likely to use services if they have suffered longer, had symptoms of greater severity and perceived disability [16].

### University Students Do Not Seek Help

A study on the health service utilization of 2785 university students at a large public university in 2005 found that for these students, who have a similar profile to the national student population in the United States (ie. in a university environment “with free access to short-term psychotherapy and basic health services”), between 37% and 84% of students with apparent mental disorders did not receive treatment [17]. This highlights the need for new models of service delivery to be targeted at this population. The findings of this study are particularly relevant in the current context as university students in Australia have access to free basic health care services and most campuses have free and convenient counseling services. A study from Norway showed that students reported a need for help, but only one-third sought help from traditional methods [18]. This further highlights the need for new pathways to care. This is an interesting finding given that 25% to 35% of health professional students reported alarming symptoms of depression [19].

### People Take Too Long to Get Help

Delays in seeking professional treatment are common, especially in those with short symptom duration and only a minority of people with disorders receive any treatment. Due to the high prevalence of depressive disorder, chronicity, early age of onset and resulting serious impairment, the World Health Organization advocates a need for early outreach and intervention programs, as well as quality assurance programs to investigate the problem of inadequate treatment [20].

The US National Comorbidity survey showed that over 80% of 15 to 54 year olds with a lifetime prevalence of Diagnostic and Statistical Manual of Mental Disorders-III-Revised (DSM-III-R) disorder eventually contact a health professional, but that it may take them more than 10 years, on average, to do so [21]. Most of these young people are unlikely to get professional help. Treatment contact delay was found to be between 6 and 14 years across a range of psychiatric disorders [22]. It was also found that only one-third of subjects with a current disorder reported contacting psychiatric services and only 16% continued this contact [23].

The Canadian National Mental Health Survey with 36,984 respondents found that only 32% of people with symptoms of mental disorder or substance dependencies saw or spoke to a health professional in the previous 12 months. They also found that teens and young adults (15 to 24 years) are least likely to use mental health services, despite a higher prevalence of mental health problems, with one survey finding that only 32% of those had talked to a health care professional in the previous 12-month period [24]. A survey of Australian youth found that they are similarly unlikely to seek professional help [25].

### Improving Screening and Treatment

One of the strategies for overcoming barriers to seeking help for depression and anxiety is to improve screening and treatment [14]. Internet-based self-help programs delivering Cognitive Behavioral Therapy (CBT) to the individual and self-awareness may prove to be a way of overcoming this barrier as it serves to provide an accurate screening tool, as well as educating the user about depression and related disorders.

### Internet Self-Help

The availability of these services is one part of the spectrum of mental health service delivery. Initiatives to improve Internet service efficacy includes access and use of services. Assessing the current awareness, access, and use of such resources in order to inform health promotion strategies is the first step in this process.

### Aim

The aim of this prospective study is to investigate awareness, access and use of Internet self-help in the university student population.

## Methods

### Awareness, Access and Use of Internet Self-Help in University Aged Students

Ethics approval from the University of Sydney and the University of Technology, Sydney (UTS) was granted in order to conduct the Awareness, Access and Use Prospective study. Deans of the UTS Faculties of Information Technology, Education, Law, Nursing and Science were supportive. From the University of Sydney, the Deans approached were from the Faculties of Science and Health Sciences; both were also supportive. Students from the Faculty of Science (at both universities) and from the Faculty of Health Sciences (University of Sydney) had the highest response rates. A pilot study was conducted in order to test cost, response rate and the feasibility of administering the large-scale prospective study at 3 time points across each university semester. The study ran for 2 years (equivalent to 4 university student semesters).

### Data Collection

To test the aims of the study, a questionnaire was administered at 3 time points over a 2-month period to assess student responses at baseline (T1) and any subsequent changes (T2 and T3). The questionnaire asked students about their Internet access, the amount of time they spent online and whether it is for work and/or study or leisure purposes. It then progressed to Internet self-help in general and then about how familiar they were with or whether they had accessed or used an Internet self-help website for emotional health. The Awareness, Access and Use Questionnaire also asked them what they would consider important in a website, credibility, as well as what they thought of free versus fee-based self-help websites. This was the first part of a two-part questionnaire. The first part consisted of questions related to the Awareness, Access and Use of Internet self-help (this study), the second part is the Center for Epidemiological Studies-Depression Questionnaire (CES-D or CESD), which was part of another larger CyberPsychiatry Study [26]. The findings of that study are reported elsewhere [26].

The two-part questionnaire was administered at 3 time points, one month apart, during the university semester. The study was therefore designed to be conducted near the beginning, the middle and the end of the semester, but avoided the exam period. Questionnaires were distributed as close as possible to weeks 2, 6 and 10 of the 12-week university semester.

Once baseline was complete (T1), the questionnaire was next distributed at T2. At T2 students who scored greater than or equal to 16 on the CESD were randomized into one of two groups. The intervention group consisted of students being randomized to the self-help website, which in this study was Moodgym [27]. The control group students were randomized to an information package, which was a psychoeducational pamphlet accessed through the Internet via a hyperlink [28]. Those results are reported elsewhere [26]. It is worth noting that completing the CESD questionnaire at the same time and being randomized to either group may have improved their awareness, access and use over the study period (T2 and T3).

### Measures

The datasheet design was pilot tested in focus groups to gain student feedback to improve the questionnaire. Suggestions were incorporated and the final questionnaire was then used as the study instrument. Questions used to facilitate this focus group were adapted from Morgan and Kruger’s Focus Group Kit and Frazer and Lawley’s Questionnaire Design [29,30]. The final survey instrument is shown in Multimedia Appendix 1.

### Statistical Analysis

Descriptive statistics were obtained using data collected and input into a Microsoft Access database, cleansed using Microsoft Excel and analyzed using SPSS statistical software.

## Results

The number of participants in the prospective Awareness, Access and Use of Internet Self-Help study after exclusions was 2970. Of those, 90.61% (2691/2970) were 18 to 24 years of age, of whom 79.75% (2146/2691) completed the questionnaire at T1, where over half (57.88%, 1242/2146) were female. Most of the students surveyed (81.97%, 1759/2146) were from UTS, with the Faculties of Information Technology, Science and Health Sciences having the highest numbers of participants.

**Figure 1:**
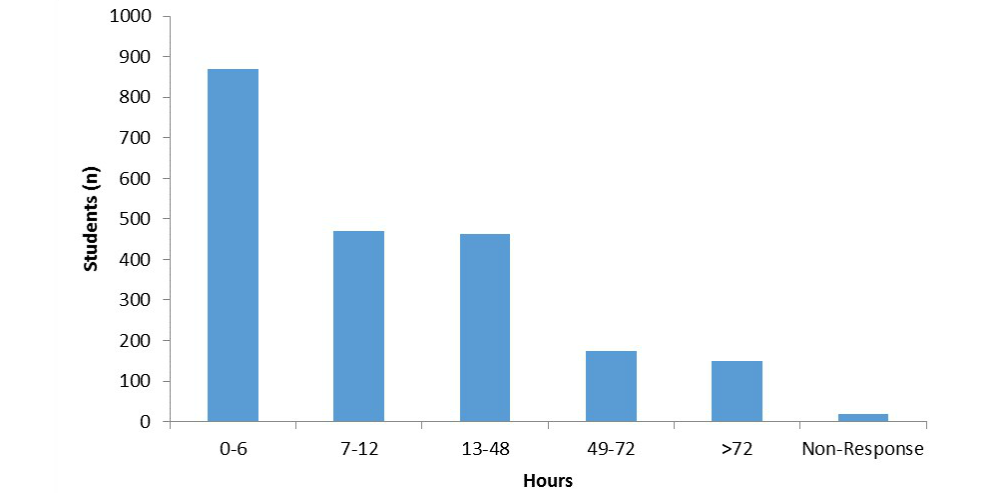
Number of hours per week students spend online for personal use.

The majority of students (77.26%, 1658/2146) had Internet access 24 hours a day, 7 days per week. Large proportions of students spent many hours on the Internet for personal use (Figure 1).

**Figure 2:**
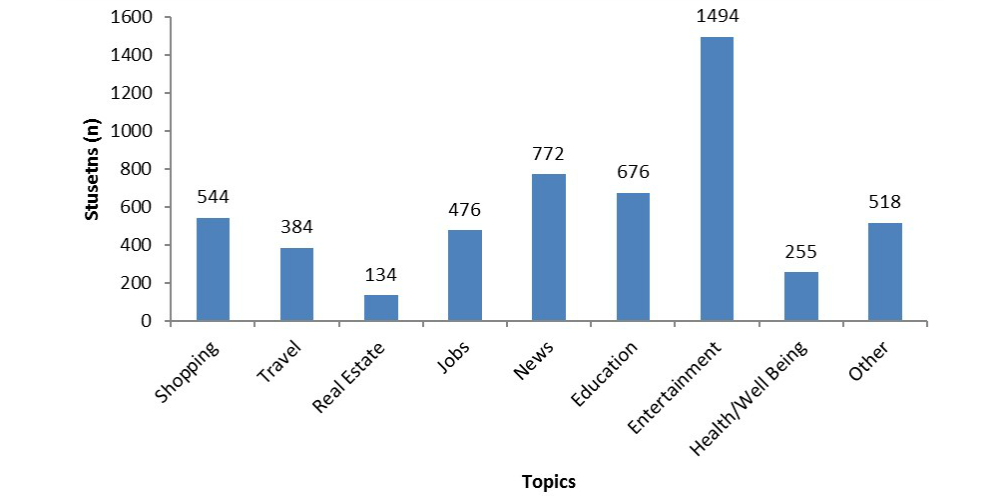
Topics browsed by students online.

When asked about browsing behavior, 70% (1494/2146) of students reported using the Internet for entertainment, whereas 12% (255/2146) used it for health and well-being. As more than one choice was enabled on the Awareness, Access and Use Questionnaire, browsing topics do not equate to 100% (Figure 2).

When asked about their familiarity with self-help websites, 166 students named one, of which most (12.7%, 21/166) named beyondblue, 7 (4.2%, 7/166) named Kidshelpline, 3 (1.8%, 3/166) named MoodGym and 7 (4.2%, 7/166) named Reachout. In using health and well-being websites, 17 students (15.3%, 17/111) had browsed them before, 23 (20.7%, 23/111) had visited one for emotional health and 12 (10.8%, 12/111) had done so in the last 6 months. When asked whether they would pay to use a self-help website, 62.63% (1344/2146) of students said they would not use it unless it was for free. Detailed results are reported elsewhere [26]. It was found that 221 (10.30%, 221/2146) students had visited a self-help website for emotional health.

### Website Qualities

Most responders always (21.48%, 461/2146), often (19.90%, 427/2146) and sometimes (14.40%, 309/2146) consider the credibility and professional authenticity (support or affiliation) of a self-help website, whilst only 3.03% (65/2146) said they never considered it. They rated self-help websites as very useful (7.18%, 154/2146) or of little use (16.31%, 350/2146), with 15 students (0.70%, 15/2146) rating them as harmful.

Most students consider user-friendliness, content and interactivity as very important in the design of a self-help website (Table 1). Therefore, websites need to contain good quality content, be user-friendly and interactive to be more likely to be accessed and used by students.

**Table 1:** Self-help website characteristics of importance to students (N=2146).

Importance	Very, n (%)	Some, n (%)	Little, n (%)	None, n (%)	Not applicable, n (%)	Non-response, n (%)
Appearance	540 (25.2)	718 (33.5)	134 (6.2)	66 (3.1)	623 (29.0)	65 (3.0)
User-friendly	1127 (52.5)	265 (12.3)	35 (1.6)	46 (2.1)	609 (28.4)	64 (3.0)
Interactivity	839 (39.1)	480 (22.4)	86 (4.0)	42 (2.0)	628 (29.3)	71 (3.3)
Content	1183 (55.1)	207 (9.6)	34 (1.6)	41 (1.9)	610 (28.4)	71 (3.3)
Screening	542 (25.3)	639 (29.8)	212 (9.9)	50 (2.3)	635 (29.6)	68 (3.2)

**Table 2:** Internet self-help website use by CESD score at time point 2 (T2) followed through time.

	Time point 1, n (%)			Time point 2, n (%)			Time point 3, n (%)		
	CESD score <16	CESD score ≥16	Total	CESD score <16	CESD score ≥16	Total	CESD score <16	CESD score ≥16	Total
Browse health/ well-being	49 (6.6)	31 (4.2)	80 (10.8)	58 (7.8)	32 (4.3)	90 (12.1)	51 (6.9)	29 (3.9)	80 (10.8)
Visited for emotional health	36 (4.8)	33 (4.4)	69 (9.3)	38 (5.1)	41 (5.5)	79 (10.6)	43 (5.8)	58 (7.8)	101 (13.6)
Visited ≥1 in last 6 months	14 (1.9)	19 (2.6)	33 (4.4)	13 (1.7)	21 (2.8)	34 (4.6)	15 (2.0)	23 (3.1)	38 (5.1)

### Use Over Time

Of the 2691 students (18-24 year olds) in the prospective study, 27.61% (743/2691) completed T1, T2 and T3. The Internet use questions and how they changed over time are shown in Table 2.

## Discussion

### Principal Findings

Overall, students indicated an awareness of and willingness to use Internet self-help. Increased activity in public health campaigns may have led to a heightened awareness of depression and related issues [31]. Previous studies have shown that a higher level of education predicted treatment-seeking behavior [9] and the results of this study may support those findings as university students have shown a willingness to access Internet self-help. This study took a selected population survey approach and did not address all 18 to 24 year old treatment-seeking behavior.

As 11.88% (255/2146) of students had accessed health and well-being specifically, this was an important finding as a previous study has shown that half (47%) of consumers said that health information on the Internet influenced their treatment decisions, prepared them to ask more questions and influenced their further help-seeking. This is important because Internet self-help may be a first step on the pathway to care for those who would otherwise not seek help or would take too long to do so. As a way of reaching out to young people and engaging them, Internet self-help for depression is in line with World Health Organization advice for early outreach and intervention programs.

Effective Internet-delivered CBT programs are not going to help people if they are not being used. The findings of this study show that as awareness through exposure increased, so did Internet access and use. It showed that approximately 1 in 10 students browse the Internet for health, well-being and emotional health and of those, half the students re-visit these websites regularly. As prevalence of depression is 20% in this population, it is promising that 10% of students are seeking health information on the Internet. Internet delivered CBT may therefore be an effective way of reaching out to those in need.

This study is not without its limitations. It would have been ideal to conduct the survey online as the topic is about online self-help; however, paper-based face-to-face surveys were chosen as they were expected to give a higher response rate. It is doubtful that an online survey would have obtained the same level of participation [26].

It is important for self-help Web surfers of low mood to be aware of the freely-available quality criteria in rating a website because the majority of students always (21.48%, 461/2146), often (19.90%, 427/2146) or sometimes (14.40%, 309/2146) consider the credibility of a self-help website, which is encouraging.

### Conclusions

More students reported visiting self-help websites as their awareness of such resources increased. This is an indication that increased awareness of depression and related resources seems to increase use of such resources. It is important to increase public awareness so that targeted strategies, including those for young people will be more widely utilized. As this study shows that a proportion of students are aware of Internet self-help and that many students do access these resources or access them regularly, these early findings may be used as a basis for further research on help-seeking behavior.

## Appendix

Multimedia Appendix 1 Internet Self-Help Awareness, Access and Use Questionnaire.

## Abbreviations

CBT: Cognitive Behavioral Therapy
CESD: Center for Epidemiological Studies -Depression
UTS: University of Technology, Sydney
