# Supplementary material for: Awareness, Access and Use of Internet Self-Help Websites for Depression by University Students
Source: JMIR Ment Health. 2016 Oct 27;3(4):e48. doi: 10.2196/mental.5311 (PMC5104907; doi:10.2196/mental.5311)
Supplement: Multimedia Appendix 1 [file mental_v3i4e48_app1.pdf]

CyberPsychiatry: Internet Self-Help Sites for Depression and Related Disorders
Consent Form

Surname	First Name

I give consent to my participation in the above titled research project. In giving my consent I acknowledge that:

1. The procedures required for the project have been explained to me, and any questions I have about the project have been answered to my satisfaction;
2. I have read the Subject Information Statement and have been given the opportunity to discuss the information and my involvement in the project with family and /or friends.
3. I am aware of the risks and inconveniences associate with the project;
4. I understand that I can withdraw from the study at any time, without affecting my treatment or my relationships with the researcher(s) now or in the future.
5. I understand that my involvement is strictly confidential and no information about me will be used in any way which reveals my identity.
6. I understand that in the event that my responses on the Depression measure indicate any clinical concern, I will be advised to either contact University Health Services or one of the study investigators.

Signed: ____________________

Self- help Website Awareness, Access and Use

In this study, Self-Help is taken to mean independently using material or therapy to help yourself with a particular issue witho seeking help from a doctor, counsellor or other health professional. For the following questions, please tick the most appropri response. If you do not wish to participate, please tick here ☐ and state your reason(s) in the comments section.

Student ID ☐☐☐☐☐☐☐☐ Date of Birth (dd/mm/yyyy) ☐☐ / ☐☐ / 1 9 ☐☐ Today's Date (dd/mm/yyyy) ☐☐ / ☐☐ / 2 0 0 ☐

e-mail address:

Since the previous time point, I was randomly assigned to:

- ☐ Website
- ☐ Information Package
- ☐ I was not randomly assigned an activity

If you were randomised, did you complete the assigned task(s)?

- ☐ Yes
- ☐ No

Gender: ☐ Male ☐ Female

University:

- ☐ UTS
- ☐ Sydney

Faculty:

- ☐ Information Technology
- ☐ Medicine
- ☐ Education
- ☐ Law
- ☐ Nursing
- ☐ Science
- ☐ Health Sciences

This is to see if and how your answers have changed since the previous time point:

1) How much Internet Access do you personally have in general (you may indicate more than one)?

- ☐ 24hours/7days and/or wireless
- ☐ at home only (nights)
- ☐ at work/Uni (daytime)
- ☐ Other ……………………………
- ☐ None

2) How much time do you spend per week on the Internet for personal (not work or study) use?

- ☐ 0-6 hours (half a day per week)
- ☐ 7-12 hours (about one day per week)
- ☐ 13-48 hours (3 days per week)
- ☐ 49-72 hours (3 days per week)
- ☐ over 72 hours

Actual Hours (if you know):_____

3) What topic most interest you when browsing (you may insert preference numbers instead of ticks)?

- ☐ Shopping
- ☐ Education
- ☐ Travel
- ☐ Entertainment
- ☐ Real estate
- ☐ Health/Well-Being Sites
- ☐ Jobs
- ☐ Other………………
- ☐ News/Current Affairs

4) Which search engine(s) do you use most (you may indicate more than one)?

- ☐ Netscape
- ☐ Alta-Vista
- ☐ Lycos
- ☐ Yahoo
- ☐ Google
- ☐ MSN
- ☐ Metacrawler
- ☐ Other:………………..

**5) What type of media have you ever used for self-help** (Self-Help meaning independently using material or therapy to help yourself with a particular issue without seeking help from a doctor, counsellor or other health professional. (eg exercise video). You may indicate more than one)?<br>☐ Book ☐ Telephone Helpline<br>☐ CD ☐ Internet<br>☐ Video ☐ None<br>☐ Seminar or Discussion Group ☐ Other………………………	**6) Are you familiar with self-help websites in *general*?**<br>☐ Yes ☐ No<br>If yes, which are you familiar with:<br>____________________<br>____________________
**7) How familiar are you with self-help websites for *emotional health*?**<br>☐ I have visited one or more in the past<br>☐ I can name one example……………………………<br>☐ I have heard of them but never used one<br>☐ I have never heard of them	**8) How often in the last 12 months have you visited a self-help website for *emotional* health?**<br>☐ Never Visited ☐ Once in 3 months<br>☐ Only visited once ☐ Monthly<br>☐ Once in 12 months ☐ Weekly or Fortnightly<br>☐ Once in 6 months ☐ Daily<br>If never, please skip to **CES-D** ***over the page.***

---

9) In your experience, how would you describe self-help websites in terms of their usefulness?

☐ very useful	☐ of little use	☐ no use	☐ harmful because:…………..	☐ not applicable

In a self-help website, how important would you consider the following? Please tick:

	very important	of some importance	of little importance	no importance
**10) Appearance (look)**				
**11) How easy it is to use**				
**12) Interactivity (meaning it engages the user)**				
**13) Content**				
**14) Screening facilities (eg. Online tests)**				

**15) Do you consider the credibility of the website and the professional authenticity (support or affiliation) of the site host?**<br>☐ Always<br>☐ Often but not always<br>☐ Sometimes<br>☐ Never<br>☐ Not Applicable	**16) How much would you be prepared to pay to use a self-help website?**<br>☐ I would not use it unless it was for free<br>☐ \$0-\$20 per year<br>☐ \$21-\$50 per year<br>☐ \$51-\$100 per year<br>☐ \$101-\$200 per year<br>☐ Over \$200 per year

Please note down any additional comments or observations:
